# Extreme Diversity of Mycoviruses Present in Single Strains of Rhizoctonia cerealis, the Pathogen of Wheat Sharp Eyespot

**DOI:** 10.1128/spectrum.00522-23

**Published:** 2023-07-12

**Authors:** Wei Li, Haiyan Sun, Shulin Cao, Aixiang Zhang, Haotian Zhang, Yan Shu, Huaigu Chen

**Affiliations:** a Institute of Plant Protection, Jiangsu Academy of Agricultural Sciences, Nanjing, Jiangsu, China; b Jiangsu Co-Innovation Center for Modern Production Technology of Grain Crops, Yangzhou University, Yangzhou, Jiangsu, China; Changchun Veterinary Research Institute

**Keywords:** *Rhizoctonia cerealis*, wheat sharp eyespot, mycovirus, diversity

## Abstract

Rhizoctonia cerealis is the pathogen of wheat sharp eyespot, which occurs throughout temperate wheat-growing regions of the world. In this project, the genomes of viruses from four strains of *R. cerealis* were analyzed based on Illumina high-throughput transcriptome sequencing (RNA-Seq) data. After filtering out reads that mapped to the fungal genome, viral genomes were assembled. In total, 131 virus-like sequences containing complete open reading frames (ORFs), belonging to 117 viruses, were obtained. Based on phylogenetic analysis, some of them were identified as novel members of the families *Curvulaviridae*, *Endornaviridae*, *Hypoviridae, Mitoviridae*, *Mymonaviridae*, and *Phenuiviridae,* while others were unclassified viruses. Most of these viruses from *R. cerealis* were significantly different from the viruses already reported. We propose the establishment of a new family, *Rhizoctobunyaviridae*, and two new genera, *Rhizoctobunyavirus* and *Iotahypovirus*. We further clarified the distribution and coinfection of these viruses in the four strains. Surprisingly, 39 viral genomes of up to 12 genera were found in strain R1084. Strain R0942, containing the fewest viruses, also contained 21 viral genomes belonging to 10 genera. Based on the RNA-Seq data, we estimated the accumulation level of some viruses in host cells and found that the mitoviruses in *R. cerealis* generally have very high accumulation. In conclusion, in the culturable phytopathogenic fungus *R. cerealis*, we discovered a considerable diversity of mycoviruses and a series of novel viruses. This study expands our understanding of the mycoviral diversity in *R. cerealis* and provides a rich resource for the further use of mycoviruses to control wheat sharp eyespot.

**IMPORTANCE** Rhizoctonia cerealis is a binucleate fungus that is widely distributed worldwide and can cause sharp eyespot disease in cereal crops. In this study, 131 virus-like sequences belonging to 117 viruses were obtained based on analysis of high-throughput RNA-Seq data from four strains of *R. cerealis*. Many of these viruses were novel members of various virus families, while others were unclassified viruses. As a result, a new family named *Rhizoctobunyaviridae* and two new genera, *Rhizoctobunyavirus* and *Iotahypovirus*, were proposed. Moreover, the discovery of multiple viruses coinfecting a single host and the high accumulation levels of mitoviruses have shed light on the complex interactions between different viruses in a single host. In conclusion, a significant diversity of mycoviruses was discovered in the culturable phytopathogenic fungus *R. cerealis*. This study expands our understanding of mycoviral diversity, and provides a valuable resource for the further utilization of mycoviruses to control wheat diseases.

## INTRODUCTION

Viruses are the most abundant and diverse entities on earth and exist in almost all known cellular life (1). Because of the rapid development of high-throughput sequencing and bioinformatics analysis technology, meta-virome studies can identify many viral genomes from a specific environment or biological population samples, and a large number of new viruses have recently been discovered from soil, water, ocean, intestine, invertebrate, and mammalian samples (2–4). Mycoviruses are common in major filamentous fungi, and some of them can reduce the pathogenicity of their host fungi, providing new resources for the control of fungal diseases in plants and animals (5–9).

Recent research has shown that the DNA mycovirus *Sclerotinia sclerotiorum* hypovirulence-associated DNA virus 1 (SsHADV-1) can convert its host, Sclerotinia sclerotiorum, from a typical necrotrophic pathogen to a beneficial endophytic fungus (10). It is not unique in this ability; the double-stranded-RNA (dsRNA) mycovirus *Pestalotiopsis theae* chrysovirus 1 (PtCV1) can also convert pathogenic Pestalotiopsis theae to an endophytic fungus, and the presence of PtCV1 confers high resistance against virulent *P. theae* strains to host plants (11). The effects of mycoviruses on controlling the endophytic traits of host fungi suggest that viruses may play a special role in ecosystems composed of mycoviruses, pathogenic fungi, and plants. These studies showed broad prospects for the exploitation and utilization of mycovirus resources.

*Rhizoctonia* is a form genus in the family *Ceratobasidiaceae*, which contains two genera, *Ceratobasidium* and *Thanatephorus* (12–14). The diversity of mycoviruses in Rhizoctonia solani (teleomorph, Thanatephorus cucumeris) has been widely reported (15–19). Rhizoctonia cerealis (teleomorph, Ceratobasidium cereale), the pathogen of wheat sharp eyespot, is widely distributed in temperate wheat-growing regions worldwide and causes major wheat yield losses (20). We previously reported a mitovirus named *Rhizoctonia cerealis* mitovirus (RcMV1) and 22 endornaviruses from *R. cerealis* (21–23).

Recently, we obtained a chromosome-level draft genome of *R. cerealis* (24), facilitating omics research on this fungus and the mycoviruses it harbors. In this study, the genomes of mycoviruses in four strains of *R. cerealis* were analyzed based on high-throughput transcriptome sequencing (RNA-Seq) data. This project aimed to elucidate the diversity, evolution, and spread of mycoviruses in different strains of *R. cerealis*.

## RESULTS

### Genome sequence assembly and confirmation.

After several iterations of assembly, a total of 131 nearly complete viral genome sequences of 117 viruses were identified in the four *R. cerealis* strains (Table 1). Generally, it is very difficult to determine the genomic segment number of a new virus. In this study, based on the segment numbers established for previously characterized related viruses, the first BLAST hit fully characterized in its genome was monosegmented, except for 14 orthocurvulaviruses with two segments. Among these viruses, RcMV1 in R1084 and 20 endornaviruses in R0928, R0942, R1084, and R10125 have been reported previously (22, 23).

**TABLE 1:** Information on viruses identified in the four *R. cerealis* strains

Phylum	Order	Family (genome type)	Genus or group	Virus name	Abbreviation	Host strain	Sequence length (nt)
*Duplornaviricota*	*Ghabrivirales*	Unclassified (dsRNA)	Megabirnavirus-like virus	*Rhizoctonia cerealis* megabirnavirus-like virus-0928–1	RcMBLV-0928–1	R0928	10,725
				*Rhizoctonia cerealis* megabirnavirus-like virus-0928–2	RcMBLV-0928–2	R0928	8,648
				*Rhizoctonia cerealis* megabirnavirus-like virus-0928–3	RcMBLV-0928–3	R0928	8,626
*Kitrinoviricota*	*Hepelivirales*	Unclassified [(+)ssRNA]	Alpha-like virus	*Rhizoctonia cerealis* alphavirus-like virus-0928–1	RcALV-0928–1	R0928	8,427
				*Rhizoctonia cerealis* alphavirus-like virus-0942–1	RcALV-0942–1	R0942	8,402
				*Rhizoctonia cerealis* alphavirus-like virus-1084–1	RcALV-1084–1	R1084	8,440
				*Rhizoctonia cerealis* alphavirus-like virus-10125–1	RcALV-10125–1	R10125	8,414
			Beny-like virus	*Rhizoctonia cerealis* beny-like virus-0928–1	RcBeLV-0928–1	R0928	12,336
				*Rhizoctonia cerealis* beny-like virus-1084–1	RcBeLV-1084–1	R1084	12,282
				*Rhizoctonia cerealis* beny-like virus-10125–1	RcBeLV-10125–1	R10125	12,475
				*Rhizoctonia cerealis* beny-like virus-10125–2	RcBeLV-10125–2	R10125	10,701
	*Tymovirales*		Tymo-like virus	*Rhizoctonia cerealis* tymo-like virus-0928–1	RcTyLV-0928–1	R0928	7,888
				*Rhizoctonia cerealis* tymo-like virus-0942–1	RcTyLV-0942–1	R0942	7,903
				*Rhizoctonia cerealis* tymo-like virus-1084–1	RcTyLV-1084–1	R1084	7,911
				*Rhizoctonia cerealis* tymo-like virus-10125–1	RcTyLV-10125–1	R10125	7,892
	*Martellivirales*	*Endornaviridae* [(+)ssRNA]	*Alphaendornavirus*	*Rhizoctonia cerealis* endornavirus-0928–1	RcEV-0928–1	R0928	20,866
				*Rhizoctonia cerealis* endornavirus-0928–2	RcEV-0928–2	R0928	20,602
				*Rhizoctonia cerealis* endornavirus-0928–3	RcEV-0928–3	R0928	21,771
				*Rhizoctonia cerealis* endornavirus-0928–4	RcEV-0928–4	R0928	20,927
				*Rhizoctonia cerealis* endornavirus-0928–5	RcEV-0928–5	R0928	17,345
				*Rhizoctonia cerealis* endornavirus-0942–2	RcEV-0942–2	R0942	17,824
				*Rhizoctonia cerealis* endornavirus-0942–3	RcEV-0942–3	R0942	19,710
				*Rhizoctonia cerealis* endornavirus-0942–4	RcEV-0942–4	R0942	20,901
				*Rhizoctonia cerealis* endornavirus-1084–1	RcEV-1084–1	R1084	20,773
				*Rhizoctonia cerealis* endornavirus-1084–2	RcEV-1084–2	R1084	19,974
				*Rhizoctonia cerealis* endornavirus-1084–3	RcEV-1084–3	R1084	20,896
				*Rhizoctonia cerealis* endornavirus-1084–4	RcEV-1084–4	R1084	20,869
				*Rhizoctonia cerealis* endornavirus-1084–5	RcEV-1084–5	R1084	19,717
				*Rhizoctonia cerealis* endornavirus-1084–6	RcEV-1084–6	R1084	17,495
				*Rhizoctonia cerealis* endornavirus-1084–8	RcEV-1084–8	R1084	16,383
				*Rhizoctonia cerealis* endornavirus-1084–9	RcEV-1084–9	R1084	15,480
				*Rhizoctonia cerealis* endornavirus-10125–2	RcEV-10125–2	R10125	17,274
			*Betaendornavirus*	*Rhizoctonia cerealis* endornavirus-0942–1	RcEV-0942–1	R0942	17,148
				*Rhizoctonia cerealis* endornavirus-1084–7	RcEV-1084–7	R1084	17,106
				*Rhizoctonia cerealis* endornavirus-10125–1	RcEV-10125–1	R10125	17,181
*Lenarviricota*	*Cryppavirales*	*Mitoviridae* [(+)ssRNA]	*Duamitovirus*	*Rhizoctonia cerealis* duamitovirus-0928–1	RcDMV-0928–1	R0928	3,946
				*Rhizoctonia cerealis* duamitovirus-0928–2	RcDMV-0928–2	R0928	3,872
				*Rhizoctonia cerealis* duamitovirus-0928–3	RcDMV-0928–3	R0928	3,737
				*Rhizoctonia cerealis* duamitovirus-0928–4	RcDMV-0928–4	R0928	3,436
				*Rhizoctonia cerealis* duamitovirus-0928–5	RcDMV-0928–5	R0928	2,809
				*Rhizoctonia cerealis* duamitovirus-0928–6	RcDMV-0928–6	R0928	2,674
				*Rhizoctonia cerealis* duamitovirus-0942–1	RcDMV-0942–1	R0942	3,871
				*Rhizoctonia cerealis* duamitovirus-0942–2	RcDMV-0942–2	R0942	3,723
				*Rhizoctonia cerealis* duamitovirus-0942–3	RcDMV-0942–3	R0942	3,273
				*Rhizoctonia cerealis* duamitovirus-0942–4	RcDMV-0942–4	R0942	3,180
				*Rhizoctonia cerealis* duamitovirus-0942–5	RcDMV-0942–5	R0942	2,824
				*Rhizoctonia cerealis* mitovirus	RcMV1	R1084	3,149
				*Rhizoctonia cerealis* duamitovirus-1084–2	RcDMV-1084–2	R1084	3,787
				*Rhizoctonia cerealis* duamitovirus-1084–3	RcDMV-1084–3	R1084	3,948
				*Rhizoctonia cerealis* duamitovirus-1084–4	RcDMV-1084–4	R1084	3,630
				*Rhizoctonia cerealis* duamitovirus-1084–5	RcDMV-1084–5	R1084	3,244
				*Rhizoctonia cerealis* duamitovirus-1084–6	RcDMV-1084–6	R1084	2,830
				*Rhizoctonia cerealis* duamitovirus-10125–1	RcDMV-10125–1	R10125	4,004
				*Rhizoctonia cerealis* duamitovirus-10125–2	RcDMV-10125–2	R10125	3,789
				*Rhizoctonia cerealis* duamitovirus-10125–3	RcDMV-10125–3	R10125	3,347
				*Rhizoctonia cerealis* duamitovirus-10125–4	RcDMV-10125–4	R10125	2,848

*Negarnaviricota*	*Bunyavirales*	Proposed *Rhizoctobunyaviridae* [(−)ssRNA]	Proposed *Rhizoctobunyavirus*	*Rhizoctonia cerealis* bunyavirus-0928–1 *Rhizoctonia cerealis* bunyavirus-0928–2 *Rhizoctonia cerealis* bunyavirus-0928–3	RcBYV-0928–1 RcBYV-0928–2 RcBYV-0928–3	R0928 R0928 R0928	13,512 13,459 13,450
				*Rhizoctonia cerealis* bunyavirus-0928–4	RcBYV-0928–4	R0928	13,389
				*Rhizoctonia cerealis* bunyavirus-0928–5	RcBYV-0928–5	R0928	12,874
				*Rhizoctonia cerealis* bunyavirus-0928–6	RcBYV-0928–6	R0928	12,868
				*Rhizoctonia cerealis* bunyavirus-0928–7	RcBYV-0928–7	R0928	12,147
				*Rhizoctonia cerealis* bunyavirus-0928–8	RcBYV-0928–8	R0928	11,774
				*Rhizoctonia cerealis* bunyavirus-0942–1	RcBYV-0942–1	R0942	13,071
				*Rhizoctonia cerealis* bunyavirus-0942–2	RcBYV-0942–2	R0942	13,038
				*Rhizoctonia cerealis* bunyavirus-0942–3	RcBYV-0942–3	R0942	12,859
				*Rhizoctonia cerealis* bunyavirus-1084–1	RcBYV-1084–1	R1084	13,394
				*Rhizoctonia cerealis* bunyavirus-1084–2	RcBYV-1084–2	R1084	13,000
				*Rhizoctonia cerealis* bunyavirus-1084–3	RcBYV-1084–3	R1084	13,026
				*Rhizoctonia cerealis* bunyavirus-1084–4	RcBYV-1084–4	R1084	12,981
				*Rhizoctonia cerealis* bunyavirus-1084–5	RcBYV-1084–5	R1084	12,912
				*Rhizoctonia cerealis* bunyavirus-10125–1	RcBYV-10125–1	R10125	13,531
				*Rhizoctonia cerealis* bunyavirus-10125–2	RcBYV-10125–2	R10125	13,528
				*Rhizoctonia cerealis* bunyavirus-10125–3	RcBYV-10125–3	R10125	12,997
				*Rhizoctonia cerealis* bunyavirus-10125–4	RcBYV-10125–4	R10125	12,849
		*Phenuiviridae* [(−)ssRNA]	*Lentinuvirus*	*Rhizoctonia cerealis* lentinuvirus-0928–1	RcLeV-0928–1	R0928	8,771
				*Rhizoctonia cerealis* lentinuvirus-0928–2	RcLeV-0928–2	R0928	7,080
				*Rhizoctonia cerealis* lentinuvirus-0928–3	RcLeV-0928–3	R0928	7,016
				*Rhizoctonia cerealis* lentinuvirus-0928–4	RcLeV-0928–4	R0928	7,011
	*Mononegavirales*	*Mymonaviridae* [(−)ssRNA]	*Phyllomonavirus*	*Rhizoctonia cerealis* phyllomonavirus-0928–1	RcPhV-0928–1	R0928	11,394
				*Rhizoctonia cerealis* phyllomonavirus-0928–2	RcPhV-0928–2	R0928	9,856
				*Rhizoctonia cerealis* phyllomonavirus-0928–3	RcPhV-0928–3	R0928	6,445
				*Rhizoctonia cerealis* phyllomonavirus-0942–1	RcPhV-0942–1	R0942	9,478
				*Rhizoctonia cerealis* phyllomonavirus-0942–2	RcPhV-0942–2	R0942	6,457
				*Rhizoctonia cerealis* phyllomonavirus-1084–1	RcPhV-1084–1	R1084	10,613
				*Rhizoctonia cerealis* phyllomonavirus-1084–2	RcPhV-1084–2	R1084	9,477
				*Rhizoctonia cerealis* phyllomonavirus-1084–3	RcPhV-1084–3	R1084	9,378
				*Rhizoctonia cerealis* phyllomonavirus-10125–1	RcPhV-10125–1	R10125	11,079
				*Rhizoctonia cerealis* phyllomonavirus-10125–2	RcPhV-10125–2	R10125	10,547
				*Rhizoctonia cerealis* phyllomonavirus-10125–3	RcPhV-10125–3	R10125	4,624

*Pisuviricota*	*Durnavirales*	*Curvulaviridae* (dsRNA)	*Orthocurvulavirus*	*Rhizoctonia cerealis* orthocurvulavirus-0928–1	RcOCV-0928–1	R0928	2,149; 1,778^*a*^
				*Rhizoctonia cerealis* orthocurvulavirus-0928–2	RcOCV-0928–2	R0928	2,222; 1,750
				*Rhizoctonia cerealis* orthocurvulavirus-0928–3	RcOCV-0928–3	R0928	2,285; 1,789
				*Rhizoctonia cerealis* orthocurvulavirus-0928–4	RcOCV-0928–4	R0928	2,252; 1,767
				*Rhizoctonia cerealis* orthocurvulavirus-0942–1	RcOCV-0942–1	R0942	2,238; 1,773
				*Rhizoctonia cerealis* orthocurvulavirus-0942–2	RcOCV-0942–2	R0942	2,148; 1,741
				*Rhizoctonia cerealis* orthocurvulavirus-0942–3	RcOCV-0942–3	R0942	2,192; 1,750
				*Rhizoctonia cerealis* orthocurvulavirus-1084–1	RcOCV-1084–1	R1084	2,230; 1,815
				*Rhizoctonia cerealis* orthocurvulavirus-1084–2	RcOCV-1084–2	R1084	2,225; 1,722
				*Rhizoctonia cerealis* orthocurvulavirus-1084–3	RcOCV-1084–3	R1084	2,154; 1,872
				*Rhizoctonia cerealis* orthocurvulavirus-1084–4	RcOCV-1084–4	R1084	2,015; 1,661
				*Rhizoctonia cerealis* orthocurvulavirus-10125–1	RcOCV-10125–1	R10125	2,239; 1,732
				*Rhizoctonia cerealis* orthocurvulavirus-10125–2	RcOCV-10125–2	R10125	2,238; 1,749
				*Rhizoctonia cerealis* orthocurvulavirus-10125–3	RcOCV-10125–3	R10125	2,163; 1,774
		*Hypoviridae* [(+)ssRNA]	*Zetahypovirus*	*Rhizoctonia cerealis* zetahypovirus-0928–1	RcZHV-0928–1	R0928	13,262
				*Rhizoctonia cerealis* zetahypovirus-0928–2	RcZHV-0928–2	R0928	13,292
				*Rhizoctonia cerealis* zetahypovirus-0942–1	RcZHV-0942–1	R0942	13,274
				*Rhizoctonia cerealis* zetahypovirus-1084–1	RcZHV-1084–1	R1084	13,292
				*Rhizoctonia cerealis* zetahypovirus-10125–1	RcZHV-10125–1	R10125	14,723
				*Rhizoctonia cerealis* zetahypovirus-10125–2	RcZHV-10125–2	R10125	12,321
				*Rhizoctonia cerealis* zetahypovirus-10125–3	RcZHV-10125–3	R10125	13,337
			Proposed *Iotahypovirus*	*Rhizoctonia cerealis* hypovirus-0928–1	RcHV-0928–1	R0928	20,697
				*Rhizoctonia cerealis* hypovirus-0942–1	RcHV-0942–1	R0942	20,803
				*Rhizoctonia cerealis* hypovirus-1084–1	RcHV-1084–1	R1084	20,713

Unclassified	Unclassified	Unclassified^*b*^	Putative virus	*Rhizoctonia cerealis* putative virus-1084–1	RcPuV-1084–1	R1084	6,728
				*Rhizoctonia cerealis* putative virus-10125–1	RcPuV-10125–1	R10125	6,641

a Entries with two values indicate the lengths of RNA1 and RNA2 of the orthocurvulavirus.

b The genome type of this putative virus is unknown.

We calculated the number of reads per kilobase per million mapped reads (RPKM) of each viral genome sequence based on the rRNA-depleted RNA-Seq and dsRNA-Seq data. For most viral genome sequences, the RPKM values obtained based on dsRNA-Seq data were higher than those based on rRNA-depleted RNA-Seq data (Table 2). However, for mitoviruses, higher RPKM values were obtained based on RNA-depleted RNA-Seq data (Table 2). After combining these two data sets, we calculated the depth of coverage of each viral genome sequence. Most viral genomes showed high coverage, and only 13 of them showed less than 100-fold coverage (Table 2). Seven sequences with less than 20-fold coverage were further verified by reverse transcription-PCR (RT-PCR) and Sanger sequencing to confirm that all viral genome sequences were reliable.

**TABLE 2:** Numbers of reads mapped to viral genomes, RPKM, and depth of coverage for each virus based on dsRNA-Seq and rRNA-depleted RNA-Seq data

Virus	Sequence length (nt)	dsRNA-Seq		rRNA-depleted RNA-Seq		Depth of coverage (×)
		No. of reads	RPKM	No. of reads	RPKM	
RcMBLV-0928–1	10,725	31,554	175.80	631	1.31	450.14
RcMBLV-0928–2	8,648	94,104	650.20	38	0.10	1,632.90
RcMBLV-0928–3	8,626	9,301	64.43	0	0.00	161.74
RcALV-0928–1	8,427	13,225	93.77	12	0.03	235.62
RcALV-0942–1	8,402	15,776	114.19	271	0.79	286.49
RcALV-1084–1	8,440	38,921	225.48	0	0.00	691.72
RcALV-10125–1	8,414	1,644	11.01	16	0.04	29.59
RcBeLV-0928–1	12,336	21,065	102.03	4,701	8.49	313.30
RcBeLV-1084–1	12,282	24,951	99.33	15,656	21.68	495.93
RcBeLV-10125–1	12,475	19,673	88.83	13,641	24.87	400.57
RcBeLV-10125–2	10,701	13,766	72.46	13,049	27.74	375.88
RcTyLV-0928–1	7,888	5,698	43.16	702	1.98	121.70
RcTyLV-0942–1	7,903	1,915	14.74	878	2.71	53.01
RcTyLV-1084–1	7,911	4,232	26.16	912	1.96	97.54
RcTyLV-10125–1	7,892	12,398	88.49	1,612	4.65	266.28
RcEV-0928–1	20,866	244,152	699.15	155	0.17	1,756.26
RcEV-0928–2	20,602	558,927	1,621.06	207	0.22	4,070.97
RcEV-0928–3	21,771	951,790	2,612.25	218	0.22	6,559.24
RcEV-0928–4	20,927	21,583	61.63	4	0.00	154.73
RcEV-0928–5	17,345	54,149	186.54	20	0.03	468.45
RcEV-0942–1	17,148	656,865	2,329.62	26,793	38.13	5,980.21
RcEV-0942–2	17,824	223,924	764.04	611	0.84	1,889.60
RcEV-0942–3	19,710	231,338	713.81	658	0.81	1,765.57
RcEV-0942–4	20,901	132,591	385.81	68	0.08	952.05
RcEV-1084–1	20,773	189,500	446.05	0	0.00	1,368.36
RcEV-1084–2	19,974	571,647	1,399.38	399	0.34	4,295.93
RcEV-1084–3	20,896	32,347	75.69	0	0.00	232.20
RcEV-1084–4	20,869	102,959	241.23	0	0.00	740.04
RcEV-1084–5	19,717	752,391	1,865.85	979	0.84	5,731.37
RcEV-1084–6	17,495	2,148,281	6,004.14	0	0.00	18,419.10
RcEV-1084–7	17,106	863,582	2,468.48	21,612	21.49	7,762.14
RcEV-1084–8	16,383	3,196,611	9,540.48	26,585	27.60	29,511.04
RcEV-1084–9	15,480	4,270,119	13,487.85	93,519	102.76	42,283.31
RcEV-10125–1	17,181	708,947	2,324.27	21,298	28.20	6,375.46
RcEV-10125–2	17,274	38,209	124.59	4,092	5.39	367.32
RcDMV-0928–1	3,946	28,695	434.51	1,179,470	6,660.57	45,926.19
RcDMV-0928–2	3,872	381,338	5,884.73	8,480,873	48,807.55	343,319.12
RcDMV-0928–3	3,737	21,852	349.40	1,150,309	6,859.20	47,049.55
RcDMV-0928–4	3,436	66,593	1,158.05	2,443,837	15,848.96	109,593.86
RcDMV-0928–5	2,809	23,694	504.01	880,022	6,981.10	48,258.24
RcDMV-0928–6	2,674	79,007	1,765.45	1,097,027	9,141.93	65,970.49
RcDMV-0942–1	3,871	26	0.41	372	2.35	15.42
RcDMV-0942–2	3,723	4	0.07	229	1.50	9.39
RcDMV-0942–3	3,273	66	1.23	197	1.47	12.05
RcDMV-0942–4	3,180	0	0.00	326	2.50	15.38
RcDMV-0942–5	2,824	27,814	598.99	3,993,280	34,511.81	213,585.02
RcMV1	3,149	119,841	1,860.83	1,453,445	7,850.73	74,942.17
RcDMV-1084–2	3,787	10,758	138.90	1,704,059	7,653.73	67,922.51
RcDMV-1084–3	3,948	13,284	164.52	3,189,683	13,742.14	121,693.28
RcDMV-1084–4	3,630	93,124	1,254.38	3,385,805	15,864.97	143,757.40
RcDMV-1084–5	3,244	85,329	1,286.14	7,023,255	36,824.89	328,695.31
RcDMV-1084–6	2,830	36,411	629.10	1,131,394	6,800.04	61,897.79
RcDMV-10125–1	4,004	52,245	734.97	3,196,091	18,155.67	121,690.91
RcDMV-10125–2	3,789	16,348	243.03	2,460,922	14,772.72	98,070.86
RcDMV-10125–3	3,347	152,952	2,574.07	6,554,586	44,542.73	300,606.72
RcDMV-10125–4	2,848	122,386	2,420.54	3,928,163	31,371.61	213,336.50
RcBYV-0928–1	13,512	86,828	383.97	1,917	3.16	985.18
RcBYV-0928–2	13,459	82,234	365.08	2,166	3.59	940.63
RcBYV-0928–3	13,450	75,112	333.69	1,884	3.12	858.69
RcBYV-0928–4	13,389	81,287	362.76	2,519	4.19	938.90
RcBYV-0928–5	12,874	13,432	62.34	591	1.02	163.39
RcBYV-0928–6	12,868	11,306	52.50	1,026	1.78	143.75
RcBYV-0928–7	12,147	15,718	77.32	1,095	2.01	207.62
RcBYV-0928–8	11,774	53,277	270.38	2,062	3.90	705.02
RcBYV-0942–1	13,071	14,420	67.09	3,065	5.72	200.65
RcBYV-0942–2	13,038	29,018	135.36	6,815	12.76	412.25
RcBYV-0942–3	12,859	13,527	63.98	2,784	5.28	190.27
RcBYV-1084–1	13,394	13,539	49.43	0	0.00	151.62
RcBYV-1084–2	13,000	7,113	26.75	0	0.00	82.07
RcBYV-1084–3	13,026	80,405	301.82	0	0.00	925.90
RcBYV-1084–4	12,981	68,532	258.14	0	0.00	791.91
RcBYV-1084–5	12,912	7,702	29.17	0	0.00	89.47
RcBYV-10125–1	13,531	44,474	185.14	9,883	16.61	602.58
RcBYV-10125–2	13,528	46,508	193.65	13,991	23.52	670.82
RcBYV-10125–3	12,997	10,541	45.68	6,648	11.63	198.38
RcBYV-10125–4	12,849	12,473	54.68	10,658	18.87	270.03
RcLeV-0928–1	8,771	15,620	106.41	5,792	14.72	366.18
RcLeV-0928–2	7,080	12,047	101.67	3,956	12.45	339.05
RcLeV-0928–3	7,016	9,889	84.22	3,000	9.53	275.56
RcLeV-0928–4	7,011	15,339	130.73	3,070	9.76	393.86
RcPhV-0928–1	11,394	64,726	339.43	336	0.66	856.53
RcPhV-0928–2	9,856	34,028	206.29	1,826	4.13	545.67
RcPhV-0928–3	6,445	100,264	929.55	1,133	3.92	2,359.90
RcPhV-0942–1	9,478	574	3.68	1,256	3.23	28.96
RcPhV-0942–2	6,457	90,590	853.24	3,262	12.33	2,180.24
RcPhV-1084–1	10,613	19,449	89.61	56,320	90.26	1,070.89
RcPhV-1084–2	9,477	9,385	48.42	3,430	6.16	202.83
RcPhV-1084–3	9,378	38,164	198.98	6,556	11.89	715.29
RcPhV-10125–1	11,079	72,318	367.68	17,547	36.02	1,216.69
RcPhV-10125–2	10,547	264,842	1,414.42	36,398	78.49	4,284.25
RcPhV-10125–3	4,624	81,476	992.50	739	3.64	2,667.01
RcOCV-0928–1	2,149 (RNA1)	131,729	3,662.66	127	1.32	9,203.54
	1,778 (RNA2)	160,181	5,383.09	879	11.02	13,587.74
RcOCV-0928–2	2,222 (RNA1)	77,729	2,090.22	808	8.10	5,301.78
	1,750 (RNA2)	77,049	2,630.76	36	0.46	6,607.29
RcOCV-0928–3	2,285 (RNA1)	394,717	10,321.71	168	1.64	25,922.43
	1,789 (RNA2)	224,587	7,501.12	84	1.05	18,837.70
RcOCV-0928–4	2,252 (RNA1)	315,499	8,371.08	126	1.25	21,022.98
	1,767 (RNA2)	208,255	7,042.24	485	6.12	17,719.86
RcOCV-0942–1	2,238 (RNA1)	698,913	18,992.61	2,741	29.89	47,027.75
	1,773 (RNA2)	208,284	7,144.45	1,597	21.98	17,756.43
RcOCV-0942–2	2,148 (RNA1)	70,366	1,992.28	365	4.15	4,939.32
	1,741 (RNA2)	134,428	4,695.83	2,194	30.76	11,770.99
RcOCV-0942–3	2,192 (RNA1)	209	5.80	0	0.00	14.30
	1,750 (RNA2)	168	5.84	0	0.00	14.40
RcOCV-1084–1	2,230 (RNA1)	191,037	4,188.77	208	1.59	12,864.01
	1,815 (RNA2)	128,061	3,449.96	611	5.73	10,634.05
RcOCV-1084–2	2,225 (RNA1)	406,794	8,939.61	405	3.10	27,451.62
	1,722 (RNA2)	354,961	10,079.09	181	1.79	30,935.71
RcOCV-1084–3	2,154 (RNA1)	64,222	1,457.85	4	0.03	4,472.56
	1,872 (RNA2)	56,971	1,488.06	216	1.96	4,582.29
RcOCV-1084–4	2,015 (RNA1)	482,217	11,701.49	120	1.01	35,905.98
	1,661 (RNA2)	475,507	13,997.84	348	3.56	42,973.06
RcOCV-10125–1	2,239 (RNA1)	511,373	12,864.83	83	0.84	34,264.58
	1,732 (RNA2)	730,147	23,745.58	3,353	44.03	63,524.83
RcOCV-10125–2	2,238 (RNA1)	129,605	3,261.99	294	2.99	8,706.37
	1,749 (RNA2)	12,025	387.27	48	0.62	1,035.42
RcOCV-10125–3	2,163 (RNA1)	427,463	11,131.72	722	7.59	29,693.83
	1,774 (RNA2)	392,587	12,465.30	54	0.69	33,199.63
RcHV-0928–1	20,697	18,623	53.76	14,970	16.12	243.46
RcHV-0942–1	20,803	21,482	62.80	48,957	57.44	507.90
RcHV-1084–1	20,713	14,648	34.58	21,616	17.75	262.62
RcZHV-0928–1	13,262	332,865	1,499.72	133,124	223.68	5,270.57
RcZHV-0928–2	13,292	387,096	1,740.13	152,218	255.19	6,086.15
RcZHV-0942–1	13,274	528	2.42	80	0.15	6.87
RcZHV-1084–1	13,292	435,766	1,603.01	573,747	734.20	11,392.34
RcZHV-10125–1	14,723	463,650	1,773.84	134,708	208.11	6,096.16
RcZHV-10125–2	12,321	417,752	1,909.82	372,283	687.25	9,618.15
RcZHV-10125–3	13,337	564,243	2,383.02	1,000,790	1,706.76	17,601.78
RcPuV-1084–1	6,728	8,924	64.86	0	0.00	198.96
RcPuV-10125–1	6,641	8,217	69.69	258	0.88	191.42

### Novel dsRNA viruses in the phylum *Duplornaviricota*.

**FIG 1:**
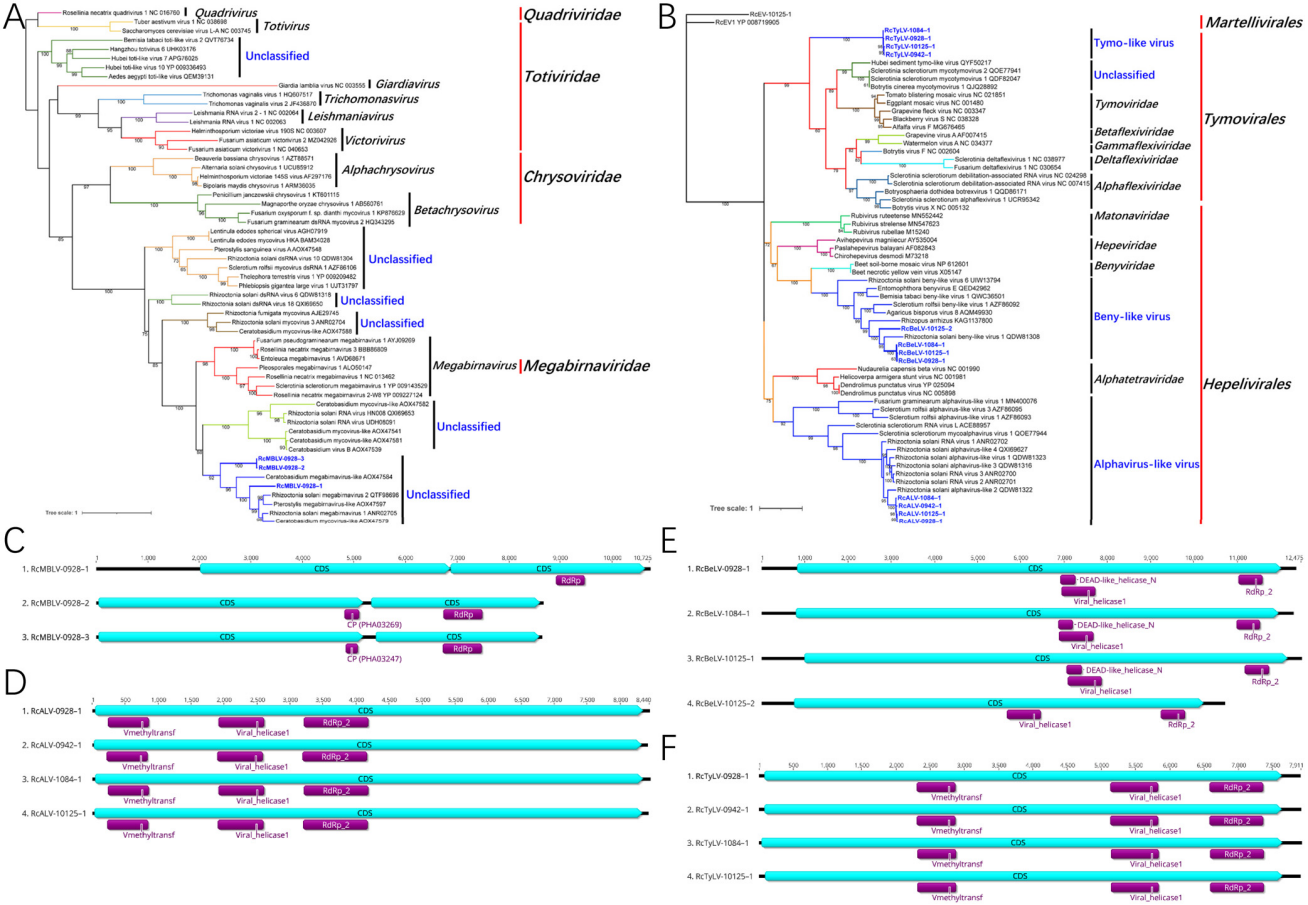
ML phylogenetic trees based on the RdRp regions and genome organization of the novel viruses identified from *R. cerealis* in the phyla *Duplornaviricota and Kitrinoviricota*. (A) Phylogenetic tree of megabirnavirus-like viruses constructed with the best-fit model LG+R5+F. (B) Phylogenetic tree of viruses in the phylum *Kitrinoviricota* constructed with the best-fit model LG+I+G4+F. (C to F) Genome organization of megabirnavirus-like viruses (C), alphavirus-like viruses (D), beny-like virus (E), and tymo-like viruses (F).

Three megabirnavirus-like dsRNA viruses in the phylum *Duplornaviricota*, order *Ghabrivirales*, were identified in strain R0928. The phylogenetic tree was reconstructed on the basis of the amino acid sequence of RNA-dependent RNA polymerase (RdRp) regions (Fig. 1A). Representative viruses in the families *Quadriviridae*, *Totiviridae*, *Chrysoviridae*, and *Megabirnaviridae* in the order *Ghabrivirales* and some viruses selected based on BLAST searches were analyzed. Many unclassified viruses closely related to the family *Megabirnaviridae* clustered on at least five branches, most of which were mycoviruses. Three mycoviruses in strain R0928 and other viruses, including *Ceratobasidium* megabirnavirus-like, *Rhizoctonia solani* megabirnavirus 1 and 2, *Pterostylis* megabirnavirus-like, and *Ceratobasidium* mycovirus-like virus, clustered on a single branch with a bootstrap support value of 92% (Fig. 1A). We named these three novel mycoviruses *Rhizoctonia cerealis* megabirnavirus-like virus 0928–1, −2 and −3, abbreviated here as RcMBLV-0928–1, −2, and −3.

All three viruses contained two open reading frames (ORFs) that were predicted to encode a putative polyprotein. The genome lengths of these three viruses ranged from 8,626 nucleotides (nt) to 10,725 nt, and all of them contained two coding sequences (CDSs), each of which was predicted to encode a putative polyprotein (Table 1; Fig. 1C). Based on a CD search, the viral RdRp domain was located in the second CDS, and two viruses contained a coat protein (CP) domain in the first CDS (Fig. 1C). The viruses in the family *Megabirnaviridae* generally have two genome segments. However, the genomes of RcMBLVs in *R. cerealis* and some other megabirnavirus-like viruses in the same clade in the phylogenetic tree had only one segment (Fig. 1A and C). The pairwise identities of the amino acid sequences of the RdRp regions of RcMBLVs compared with the viruses in the family *Megabirnaviridae* were less than 40%. Combining genome structures and phylogenetic analysis, these results indicated the identification of a new unclassified group in the order *Ghabrivirales*.

### Novel (+)ssRNA viruses in the phylum *Kitrinoviricota*.

In the four *R. cerealis* strains, we identified 32 plus-strand single-stranded-RNA [(+)ssRNA] mycoviruses in the phylum *Kitrinoviricota*. Among these viruses, 17 alphaendornaviruses and three betaendornaviruses have been reported previously (23). Based on the phylogenetic analysis, eight novel viruses belonged to the order *Hepelivirales* (Fig. 1B), four of which were named *Rhizoctonia cerealis* alphavirus-like virus-0928–1, -0942–1, -1084–1 and -10125–1, abbreviated here as RcALV-0928–1, -0942–1, -1084–1, and -10125–1. The genome lengths of these four viruses ranged from 8,402 nt to 8,440 nt. All four RcALVs contained one CDS that was predicted to encode a putative polyprotein, and Vmethyltransf, viral helicase1, and RdRp_2 domains were located in this CDS (Fig. 1D). Four novel viruses were named *Rhizoctonia cerealis* beny-like virus-0928–1, -0942–1, -1084–1 and -10125–1, abbreviated here as RcBeLV-0928–1, -0942–1, -1084–1, and -10125–1. The genome lengths of these four viruses ranged from 10,701 nt to 12,475 nt. These four RcBeLVs contained one CDS, and viral helicase1 and RdRp_2 domains were located in this CDS (Fig. 1E).

In the phylogenetic tree, the four RcALVs and other mycoviruses from R. solani, Sclerotinia sclerotiorum, Sclerotinia rolfsii, and Fusarium graminearum clustered on a branch with a bootstrap support value of 92%. This alphavirus-like virus group was the sister group to the family *Alphatetraviridae* in the order *Hepelivirales* (Fig. 1B). The four RcBeLVs and other mycoviruses clustered on a branch with a bootstrap support value of 100%, which formed the beny-like virus group. The phylogenetic position of this group was closely related to the family *Benyviridae* (Fig. 1B).

The other four novel viruses were identified as tymo-like viruses in the order *Tymovirales* (Fig. 1B). We named them *Rhizoctonia cerealis* tymo-like virus, abbreviated here as RcTyLV-0928–1, -0942–1, -1084–1 and -10125–1. The genome lengths of these four viruses ranged from 7,888 nt to 7,911 nt. They contained one CDS, and Vmethyltransf, viral helicase1, and RdRp_2 domains were located in this CDS (Fig. 1F). These four viruses from *R. cerealis* were closely related to each other, and their protein identities were more than 98%. Thus, they should be considered different variants of the same virus, RcTyLV. These four viruses clustered on a separate branch in the phylogenetic tree (Fig. 1B). Using the amino acid sequences of the RdRp region as queries in BLASTp analysis, the closest viruses identified were *Sclerotinia sclerotiorum* mycotymovirus 1 and 2 and *Botrytis cinerea* mycotymovirus 1, which belonged to an unclassified group in the order *Tymovirales*. Based on phylogenetic analysis, the tymo-like virus group was independent of the families *Alphaflexiviridae*, *Betaflexiviridae*, *Gammaflexiviridae*, *Deltaflexiviridae*, and *Tymoviridae*, which indicates the identification of a new family in the order *Tymovirales*.

### Novel (+)ssRNA viruses in the phylum *Lenarviricota*.

**FIG 2:**
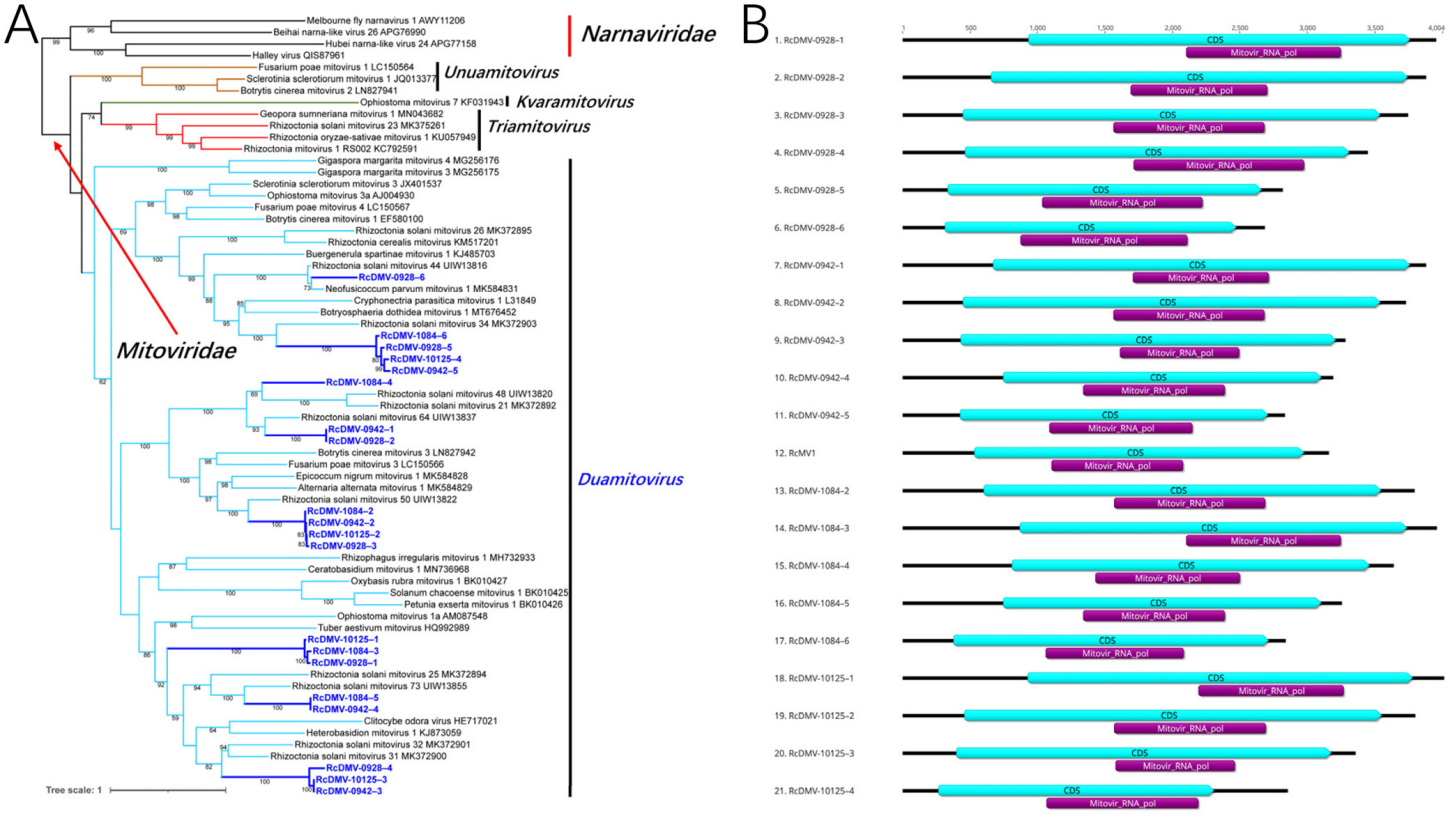
ML phylogenetic trees based on the RdRp regions and genome organization of the novel viruses identified from *R. cerealis* in the family *Mitoviridae.* (A) Phylogenetic tree of mitoviruses constructed with the best-fit model Blosum62+R6+F. (B) Genome organization of mitoviruses.

Twenty-one mitoviruses with (+)ssRNA genomes were identified in the four *R. cerealis* strains. Based on the phylogenetic analysis, all of these mitoviruses were identified as *Duamitovirus* in the family *Mitoviridae*, order *Cryppavirales*, phylum *Lenarviricota* (Fig. 2A). We named them *Rhizoctonia cerealis* duamitovirus (RcDMV). RcMV1 in strain R1084 has been reported previously (22), and we retained the name RcDMV-1084–1 for this virus (Table 1). The genome lengths of these 21 mitoviruses ranged from 2,674 nt to 4,004 nt. These RcDMVs contained one CDS, and mitovirus RNA-dependent RNA polymerase domains were located in this CDS (Fig. 2B). However, in the phylogenetic tree, the mitoviruses from *R. cerealis* did not cluster together but were dispersed in multiple clades, indicating that these RcDMVs showed rich diversity at the species level.

### Novel (−)ssRNA viruses in the phylum *Negarnaviricota*.

**FIG 3:**
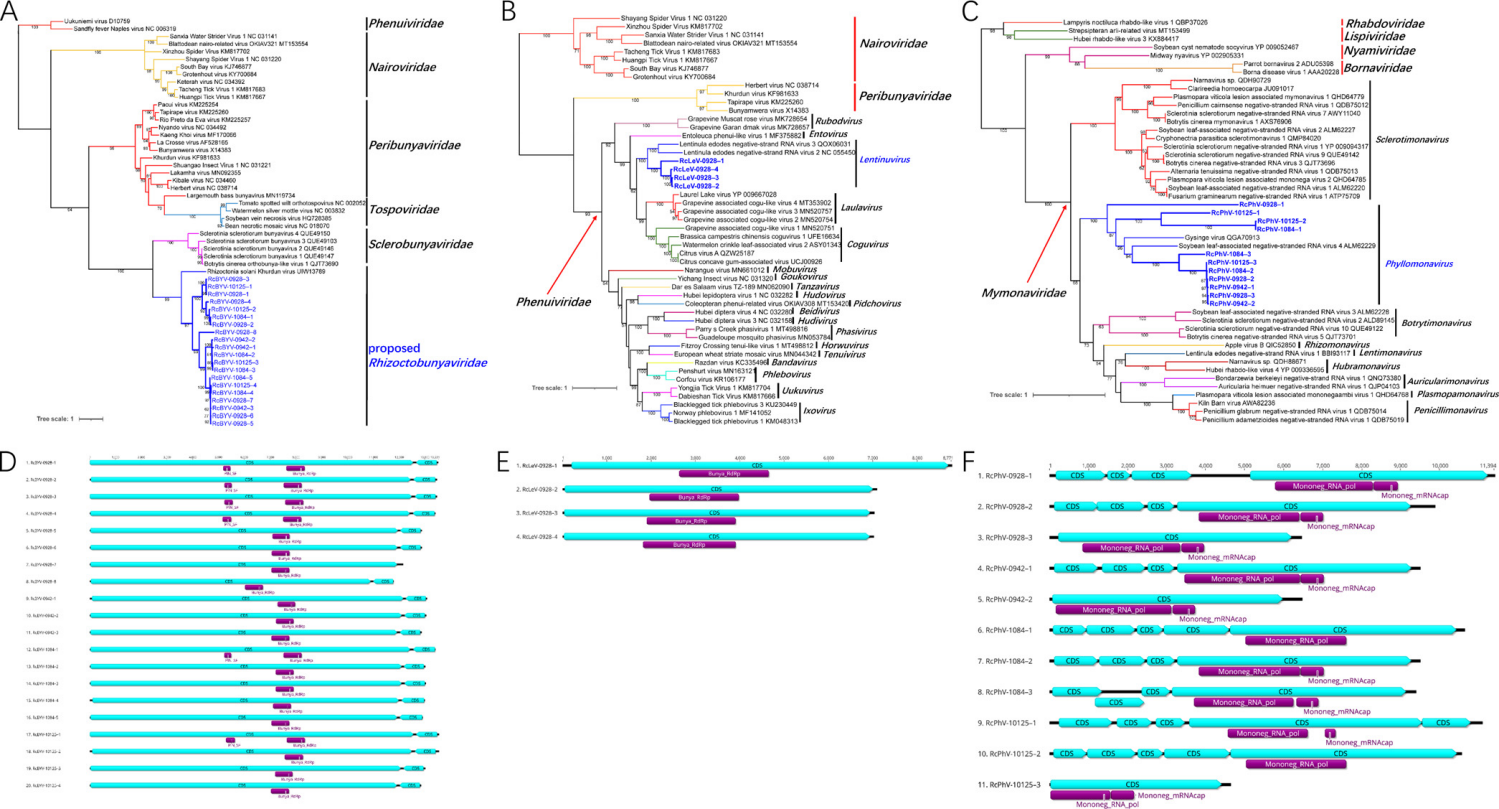
ML phylogenetic trees based on the RdRp regions and genome organization of the novel viruses identified from *R. cerealis* in the phylum *Negarnaviricota.* (A) Phylogenetic tree of bunyaviruses constructed with the best-fit model LG+I+G4+F. (B) Phylogenetic tree of lentinuviruses constructed with the best-fit model LG+I+G4+F. (C) Phylogenetic tree of phyllomonaviruses constructed with the best-fit model LG+R5. (D to F) Genome organization of bunyaviruses (D), lentinuviruses (E) and phyllomonaviruses (F).

In total, 35 novel minus-strand-ssRNA [(−)ssRNA] viruses in the phylum *Negarnaviricota* were identified in the four *R. cerealis* strains (Table 1; Fig. 3A to C). Twenty viruses were identified as novel members of the order *Bunyavirales* (Fig. 3A). These viruses were named *Rhizoctonia cerealis* bunyavirus (RcBYV). The genome lengths of these 20 RcBYVs ranged from 11,774 nt to 13,531 nt. With the exception of RcBYV-0928–7, the other 19 viruses contained 2 ORFs encompassing two CDSs, and the bunyavirus RdRp (Bunya_RdRp) domain was located in the first CDS (Fig. 3D). Some of the viruses also contained a PIN_SF domain, which belongs to a large nuclease superfamily, in the first CDS (Fig. 3D). However, no conserved domains have been found in the second CDS, and the level of identity of CDS2 among these viruses was similar to that of the RdRp domain. In the phylogenetic tree, all of the RcBYVs and *Rhizoctonia solani* Khurdun virus were clustered in a clade with a bootstrap support value of 99% (Fig. 3A). This group was independent of other families in the order *Bunyavirales* and formed sister groups with the family *Sclerobunyaviridae*, which was proposed in 2021 (25). At present, all of the viruses in this group have been identified in *Rhizoctonia* fungi, so we propose a new family, *Rhizoctobunyaviridae*, and a new genus, *Rhizoctobunyavirus*, to accommodate these viruses.

Four (−)ssRNA viruses in strain R0928 were identified as lentinuviruses in the family *Phenuiviridae* in the order *Bunyavirales* (Fig. 3B). These viruses were named *Rhizoctonia cerealis* lentinuvirus (RcLeV). The genome lengths of these four RcLeVs ranged from 7,011 nt to 8,771 nt. All of the RcLeVs contained one CDS, and the Bunya_RdRp domain was located in this CDS (Fig. 3E). Based on the phylogenetic analysis, the four RcLeVs clustered into a single clade and then clustered into a larger clade with *Lentinula edodes* negative-strand RNA virus 2 and 3 (LeNSRV2 and LeNSRV3) in the genus *Lentinuvirus* (Fig. 3B). This larger clade was independent of other genera in the family *Phenuiviridae*, with 100% bootstrap support (Fig. 3B). Therefore, we classified these four viruses from *R. cerealis* as members of the genus *Lentinuvirus*. However, LeNSRV2 and LeNSRV3 have a bipartite RNA genome. Based on the RNA2 sequences of LeNSRV2 and 3, we could not find the homologous sequences from *R. cerealis*. This means that the four RcLeVs most likely have only a monopartite RNA genome. In addition, the amino acid identity of the RdRp regions among the four RcLeVs and two LeNSRVs was less than 60%, so the possibility of establishing a new genus in the future cannot be ruled out.

The other 11 novel (−)ssRNA viruses were identified as new *Phyllomonavirus* members belonging to the family *Mymonaviridae* in the order *Mononegavirales* (Fig. 3C). These viruses were named *Rhizoctonia cerealis* phyllomonavirus (RcPhV). Most of them contained 4 or 5 CDSs (9,378 nt to 11,394 nt) in the genome. However, three incomplete virus segments were found from R0928, R0942, and R10125, and these have been temporarily named RcPhV-0928–3, RcPhV-0942–2, and RcPhV-10125–3, respectively. The lengths of these segments are 6,445 nt, 6,457 nt, and 4,624 nt; each of them contains only one CDS (Fig. 3F). Due to the limitation of the viral genome search method, we cannot confirm the complete genomes of these viruses. Some pieces may be missing from some of the genomes. All of the RcPhVs contained a Mononeg_RNA_pol domain located in the longest CDS, and some of them also had a Mononeg_mRNAcap domain located after the Mononeg_RNA_pol domain (Fig. 3F). In the phylogenetic tree, all of the RcPhVs, a Gysinge virus, and soybean leaf-associated negative-stranded RNA virus 4 were clustered in a clade with a bootstrap support value of 100% (Fig. 3C). The viruses in this clade belonged to the genus *Phyllomonavirus*, and this clade was independent of other genera in the family *Mymonaviridae* (Fig. 3C). In the clade *Phyllomonavirus*, the RcPhVs identified in *R. cerealis* clustered on at least five branches, indicating their diversity.

### Novel dsRNA and (+)ssRNA viruses in the phylum *Pisuviricota*.

**FIG 4:**
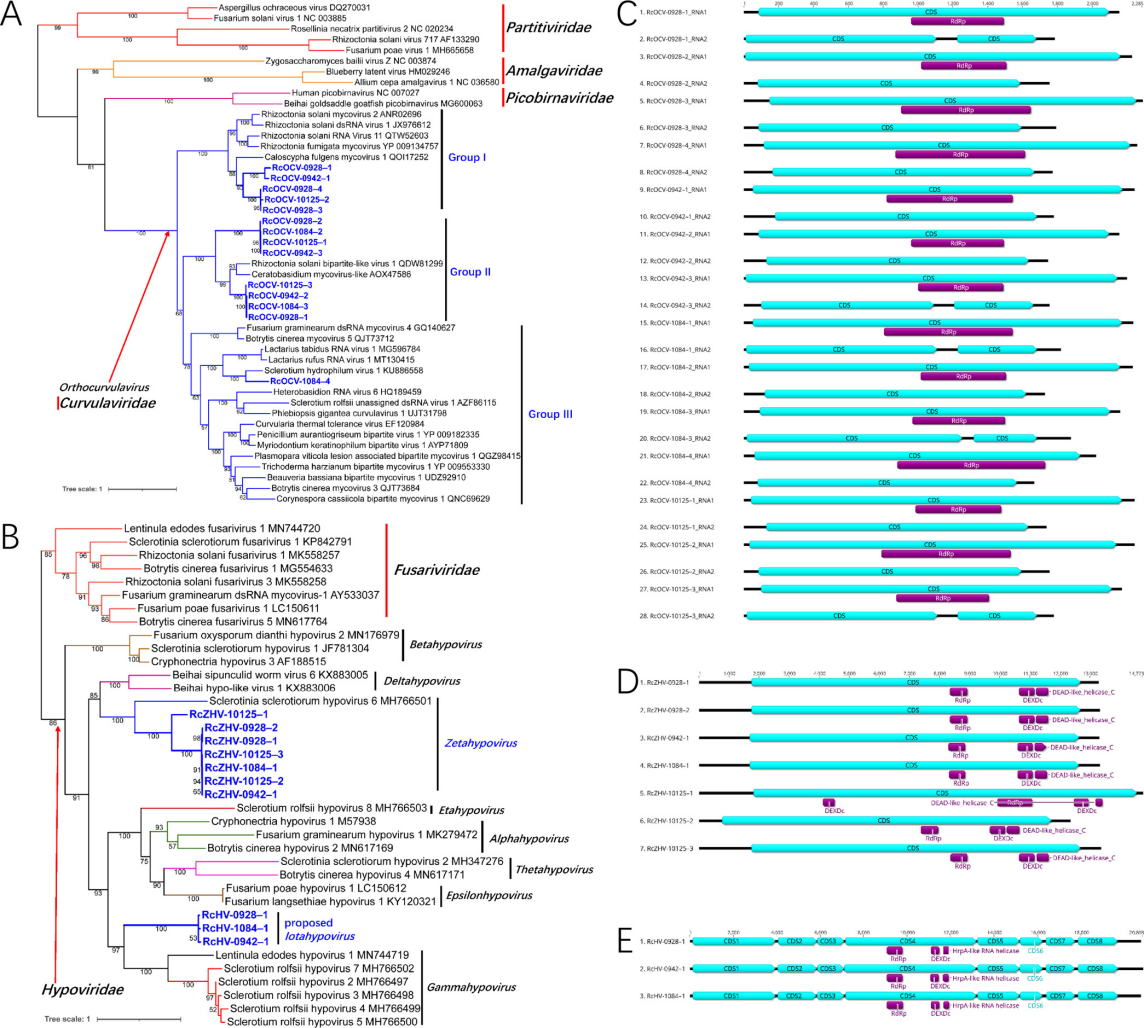
ML phylogenetic trees based on the RdRp regions and genome organization of the novel viruses identified from *R. cerealis* in the phylum *Durnavirales.* (A) Phylogenetic tree of orthocurvulaviruses constructed with the best-fit model LG+I+G4+F. (B) Phylogenetic tree of hypoviruses constructed with the best-fit model Blosum62+R3. (C to E) Genome organization of orthocurvulavirus (C), *Zetahypovirus* (D) and a proposed *Iotahypovirus* (E).

Twenty-four novel dsRNA and (+)ssRNA viruses in the four *R. cerealis* strains were identified as belonging to the phylum *Pisuviricota* (Table 1; Fig. 4A and B). Among these viruses, 14 dsRNA viruses with 2 genome segments were identified as orthocurvulaviruses in the family *Curvulaviridae*, order *Durnavirales* (Fig. 4A). These viruses were named *Rhizoctonia cerealis* orthocurvulavirus (RcOCV). The lengths of their RNA1 segments ranged from 2,015 nt to 2,285 nt, and the lengths of the RNA2 segments ranged from 1,661 nt to 1,778 nt. All of the RNA1 segments contained only one CDS, and the RdRp domain was located in this CDS (Fig. 4C). Among the 14 RNA2 segments, 5 contained two CDSs and the others contained only one CDS. There was no identifiable conserved domain within the RNA2 segment (Fig. 4C). However, since multiple RcOCV genomes could exist in the same strain, based on the number of reads (Table 2) and the partial conservation of 5′-end and 3′-end sequences, we matched RNA1 and RNA2 segments of each virus and named them (Table 1; Fig. 4C).

Currently, there is only one genus, *Orthocurvulavirus*, in the family *Curvulaviridae*. Based on the topological structure of the phylogenetic tree, the viruses in the genus *Orthocurvulavirus* could be divided into 3 groups (Fig. 4A). The hosts of viruses in groups I and II were mainly *Rhizoctonia* fungi, while the hosts of viruses in group III were various fungi. Among the 14 RcOCVs, five clustered in group I, eight clustered in group II, and only RcOCV-1084–4 was found in group III (Fig. 4A). The results of this phylogenetic analysis also provide evidence for establishing more genera in the family *Curvulaviridae*.

Based on the phylogenetic analysis, 10 (+)ssRNA viruses were related to hypoviruses (Table 1; Fig. 4B). Seven of them were identified as zetahypoviruses and were named *Rhizoctonia cerealis* zetahypovirus (RcZHV). The genome lengths of these seven RcZHVs ranged from 12,321 nt to 14,723 nt. All of the RcZHVs contained one CDS, and RdRp, DEADc and DEAD-like_helicase_C domains were located in this CDS (Fig. 4D). Three other unclassified hypoviruses were identified from strains R0928, R0942, and R1084 and were named *Rhizoctonia cerealis* hypovirus (RcHV). The genome lengths of these 3 RcHVs were 20,697 nt, 20,803 nt, and 20,713 nt, respectively, and they contained eight CDSs. RdRp, DEXDc, and HrpA-like RNA helicase domains were located in CDS4, which was the longest CDS in the genome (Fig. 4E).

At present, there are eight genera in the family *Hypoviridae*: *Alpha-* to *Thetahypovirus* (26). In the phylogenetic tree, seven RcZHVs and *Sclerotinia sclerotiorum* hypovirus 6 clustered in the clade *Zetahypovirus* with 100% bootstrap support (Fig. 4B). Notably, in contrast to the genome structure of the other six RcZHVs, the CDS of RcZHV-10125-1 contains two DEXDc domains (Fig. 4D). The three RcHVs in *R. cerealis* were closely related to each other, and their genomic identities ranged from 72.5 to 76.8%. The RcHVs clustered in a separate clade, which was independent of other genera in the family *Hypoviridae* (Fig. 4B). Based on their genome structure, which differed from those of other hypoviruses, and the results of phylogenetic analysis, we propose a new genus, *Iotahypovirus*, to accommodate these three new hypoviruses from *R. cerealis*.

### Unclassified viral genome sequences.

**FIG 5:**
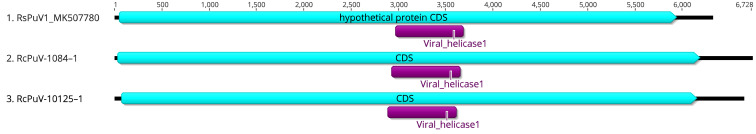
Genome organization of putative viruses identified in *R. cerealis* and RsPuV1 in R. solani.

In strains R1084 and R10125, we identified two viral genome sequences that could not be assigned to any existing clade of viruses present in the databases. The lengths of these two sequences were 6,728 nt and 6,641 nt, and each contained one CDS. We could not identify an RdRp domain in the CDS, but a viral helicase 1 domain was located within it (Fig. 5). The pairwise identity between these two sequences was 98.1%. In BLAST searches using the genome sequences or the amino acid sequences of the Viral_helicase1 domain, the first match was *Rhizoctonia solani* putative virus 1 (RsPuV1), which was identified in R. solani (19). The genome structures were similar among these 3 viral genome sequences (Fig. 5); however, the pairwise identity of the Viral_helicase1 region between the viruses from *R. cerealis* and R. solani was less than 28.5%. We named these two viruses *Rhizoctonia cerealis* putative virus, abbreviated here as RcPuV-1084–1 and RcPuV-10125–1.

### Diversity of mycoviruses present in the four strains of *R. cerealis*.

**FIG 6:**
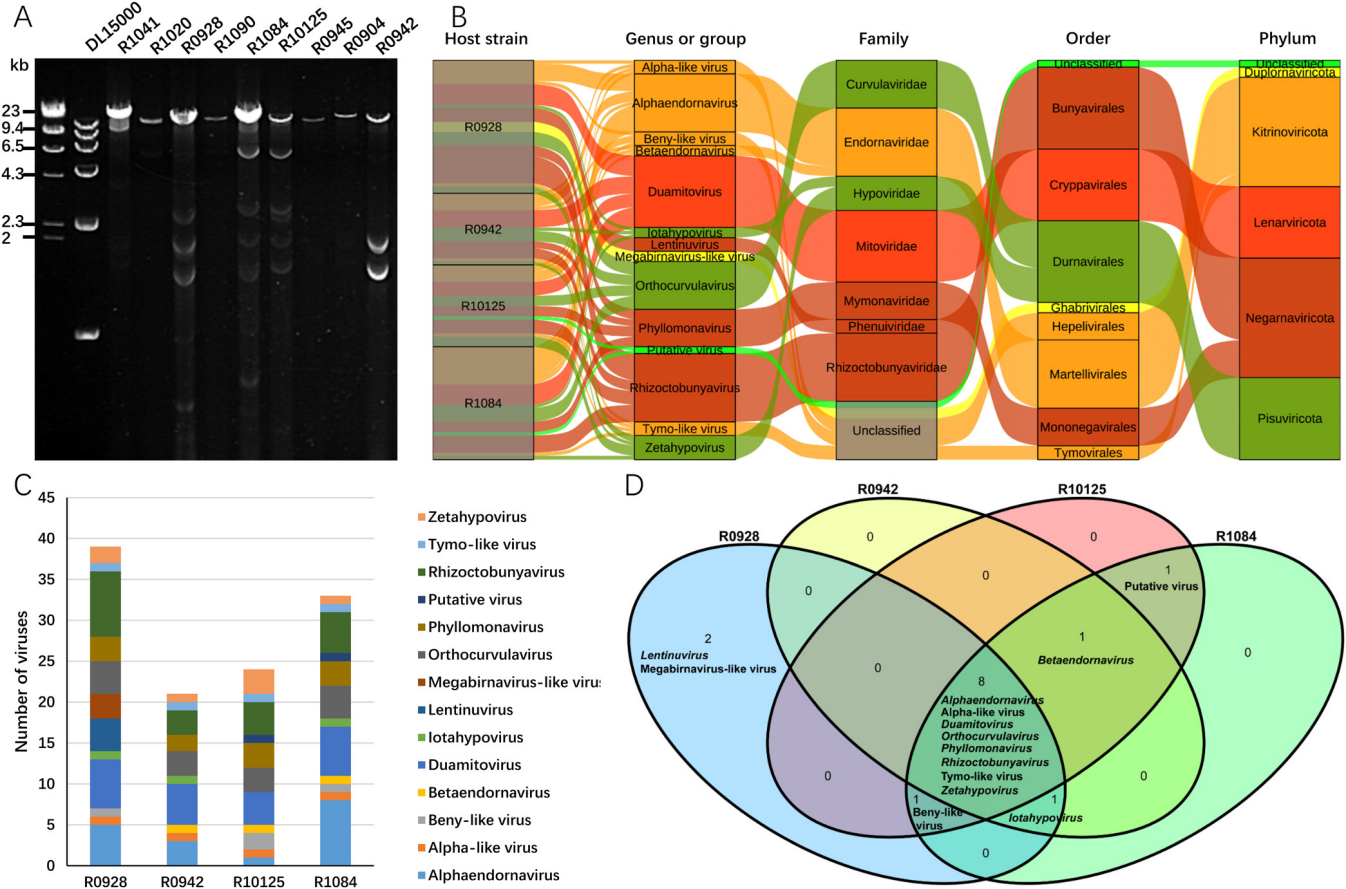
Diversity of viruses identified in *R. cerealis*. (A) Agarose gel electrophoresis of dsRNA extracted from different strains. Bands of the DL15000 marker: 15, 10, 7, 5, 2.5, and 1 kbp. (B) Sankey diagram showing the compositions of viruses from four strains. (C) Viruses belonging to different genera or groups presented in each strain. (D) Numbers and groups of shared viruses in the virome of four strains of *R. cerealis*.

The diversity of dsRNA bands in strains R0928, R0942, R1084, and R10125 was particularly rich (Fig. 6A). In these four strains, a total of 117 mycoviruses were found. These viruses belonged to at least nine known genera and five unclassified genera, seven known families and three unclassified families, and five phyla in the kingdom *Orthornavirae* (Fig. 6B). At the phylum level, (−)ssRNA viruses in the phylum *Negarnaviricota* were the most abundant, accounting for 30% of the total. With the exception of unclassified viruses, the fewest identified dsRNA viruses belonged to the phylum *Duplornaviricota*, representing only 3% of the total. At the family level, viruses of the families *Mitoviridae* and *Endornaviridae* were the most abundant, followed by the proposed new family *Rhizoctobunyaviridae* (Fig. 6B). At the genus level, the four strains contained a wide variety of viruses, among which R0928 and R1084 each contained 12 genera, R10125 contained 11 genera, and R0942 contained 10 genera. The R0928 strain harbored the greatest number of viruses, 39, while R0942 contained the fewest viruses, 21 (Fig. 6C). Viruses of eight genera could be identified in all four strains, indicating that these viruses were popular in *Rhizoctonia* fungi. Betaendornaviruses, beny-like viruses, and iotahypoviruses were identified in 3 strains, putative viruses (RcPuV) were identified in R1084 and R10125, and lentinuviruses and megabirnavirus-like viruses were identified only in R0928 (Fig. 6D).

## DISCUSSION

High-throughput sequencing has become the main method for discovering new viruses from a large number of samples (3, 4, 25). *Rhizoctonia* are soilborne fungi that mostly lack sexual reproduction, and many members of the genus *Rhizoctonia* are plant pathogens (27). In this study, we used two methods to construct cDNA sequencing libraries and combined the two resulting high-throughput sequencing data sets to assemble relatively complete genomes of 117 viruses from four *R. cerealis* strains (Table 1). The depth-of-coverage values of these viral genome sequences were generally more than 100× (Table 2). Using the RPKM value of each viral genome sequence as a measure, we compared the quality of the data obtained via the two methods. For most viruses other than mitoviruses, the dsRNA-Seq method produced higher RPKM values. Compared with the rRNA-depleted RNA-Seq method, more reads from the viral genome were obtained using the dsRNA-Seq method because a large number of nonviral RNAs were removed in the pretreatment stage. For some viruses, such as RcMBLV-0928–3, RcALV-1084–1, and RcEV-1084–1, no reads could be obtained using the rRNA-depleted RNA-Seq method but nearly complete genome sequences could be obtained using the dsRNA-Seq method (Table 2). Previously, we reported the identification of endornaviruses with extremely long genomes and 2 ORFs in the four investigated *R. cerealis* strains (23). The results of this study further demonstrate the reliability of these viral genomes and indicate that the dsRNA-Seq method may be the better choice for producing such nearly complete long viral genomes (Table 2). However, it is obvious that the RNA-Seq method would miss ssDNA viruses, and the completeness of the viral genomes also depends on the depth of sequencing.

Mitoviruses in the family *Mitoviridae* are commonly identified in filamentous fungi that have (+)ssRNA genomes of 2.3 to 3.6 kb encoding only RdRps (28). Capsidless mitoviruses localize in fungal mitochondria, within which their replication cycle is completed, consistent with the mitochondrial codon usage of fungi (29). In this study, with the exception of RcDMV-0942–1 to -0942–4, for which relatively low read numbers were obtained by both methods, unusually high RPKM values were obtained for some other mitoviruses using the RNA-depleted RNA-Seq method, although higher RPKM values were also obtained for these viruses using the dsRNA-Seq method (Table 2). dsRNA could be genomic RNA of dsRNA viruses or replicative-form dsRNA of ssRNA viruses; however, the genome of mitoviruses consists of ssRNA. The results of this work indicated that in host fungi, the accumulation levels of mitoviruses were significantly higher than those of other viruses (Table 2). Consistent with a study of *Cryphonectria parasitica* mitovirus 1 (CpMV1) in Cryphonectria parasitica strains (30), this study also demonstrated that mitochondria act as a natural protective environment, helping capsidless mitoviruses avoid interference by cytoplasmic RNA silencing.

Twenty-one duamitoviruses were discovered, and each strain contained four to six mitoviruses (Table 1), indicating that in *R. cerealis*, both the variety and accumulation level of mitoviruses were very high. At present, only a few mitoviruses, such as botrytis mitovirus 1 (BcMV1) and *Sclerotinia sclerotiorum* mitovirus 1 (SsMV1), are known to cause severe symptoms, including hypovirulence, in host fungi (31, 32). Therefore, many mitoviruses exist in *R. cerealis* mitochondria, and their biological functions are of considerable interest.

The 117 viruses with dsRNA, (+)ssRNA, and (−)ssRNA genomes identified in *R. cerealis* could be grouped into the phyla *Duplornaviricota*, *Kitrinoviricota*, *Lenarviricota*, *Negarnaviricota*, and *Pisuviricota* in the realm *Riboviria* (Table 1). Most of these viruses were sufficiently distant from those deposited in the databases to suggest that they qualify as possible new viral species or as members of new genera or even families. We propose names for these novel mycoviruses based on the name of the host strain and the number of the virus identified in the strain. This method generates names that include the host strain name to avoid the infinite expansion of numbers or the application of the same number to similar viruses by different researchers.

*Bunyavirales* is one of the largest orders of negative-strand RNA viruses in the phylum *Negarnaviricota*, containing 14 families based on the ICTV report from 2021 (https://ictv.global/taxonomy). The hosts of most bunyaviruses are vertebrates, arthropods, and plants (33). However, some newly identified bunyaviruses were discovered in the pathogenic fungus *S. sclerotiorum*, and two new families, *Mycophenuiviridae* and *Sclerobunyaviridae*, were proposed in 2021 (25). In *R. cerealis*, we identified 24 novel mycoviruses whose genomes contain a conserved Bunya_RdRp domain (Fig. 3A, B). However, the Bunya_RdRp regions of these bunyaviruses in *Rhizoctonia* fungi shared less than 26% identity with those of *Sclerobunyaviridae*, indicating that they represent a new virus lineage. Therefore, we propose a new family, *Rhizoctobunyaviridae*, and a new genus, *Rhizoctobunyavirus*, to accommodate these bunyaviruses in *Rhizoctonia* fungi. Furthermore, some additional clades with high bootstrap support also existed, indicating that more than one genus may be contained in this family (Fig. 3A). Genomes of bunyaviruses are typically tripartite, consisting of large (L), medium (M), and small (S) segments (34). However, only one segment of the genomes of bunyaviruses in *Sclerotinia* (25) and *Rhizoctonia* fungi has been found thus far. We may need more evidence to confirm the numbers of genome segments of these new bunyaviruses.

The family *Mymonaviridae* includes many mycoviruses belonging to the genera *Botrytimonaviru*s, *Lentimonavirus*, *Penicillimonavirus*, and *Sclerotimonavirus* (35). In this study, 11 RcPhVs were discovered in *R. cerealis*, which were identified as phyllomonaviruses in the family *Mymonaviridae* (Fig. 3C). This is the first report of mycoviruses in the genus *Phyllomonavirus.* The genome structures of the mycoviruses in the family *Mymonaviridae* were diverse. For example, the genome of *Sclerotinia sclerotiorum* negative-strand RNA virus 1 strain AH98 contained six CDSs (36), and that of *Botrytis cinerea* mymonavirus 1 isolate Ecan17-2 contained three CDSs (37). Similarly, eight RcPhVs contained four or five CDSs in their genomes, and the other three RcPhV genomes contained only one CDS (Fig. 3F).

Picarelli et al. (19) reported that in R. solani, there are four viral fragments, designated RsPuV 1 to 4, and the genome structures were similar to those of RcPuV-1084–1 and RcPuV-10125–1 from *R. cerealis*. We confirmed the presence of these two sequences based on the depth of coverage (greater than 190) and RT-PCR analysis (Table 2). These putative viruses contained only a Viral_helicase1 domain. Recent studies have indicated that Viral_helicase1 can undergo horizontal gene transfer between different viruses and/or bacteria (38). Although GDD amino acid triplets, the hallmark of most viral RdRps, were found in the protein sequences, there is currently no evidence suggesting that the regions in which they are located are a conserved palm domain. As a result, we cannot confirm at this time that these proteins are putative mycoviral RdRps based on these GDD amino acid triplets. They may be part of the genome of a virus for which the complete genome has not yet been obtained, or they may be fragments of multipartite virus genomes.

*R. cerealis* has no sexual reproductive stage and does not produce any asexual spores (27). This means that the viruses contained in *R. cerealis* can be transmitted only by hyphal fusion. However, it is not clear how often hyphal fusion occurs in nature. Therefore, the pattern of transmission of the viruses between *R. cerealis* strains in nature is not clear. The numbers of dsRNA gel electrophoresis bands extracted from different strains are various, and not all strains contained as many bands as R0928, R0942, R1084, and R10125 (Fig. 6A). Even among different strains collected from the same field, the number of dsRNA bands can vary, indicating that the transmission of viruses between different strains is limited. The four strains of *R. cerealis* investigated in this study were collected from geographically distant locations in three provinces in China. The virus species contained in these strains were different, and most of the genome sequences found even within the same species were different. These results also indicated that the evolution of viruses in different strains was relatively independent.

To the best of our knowledge, expansions of RNA viruses have been reported from environmental samples or nonliving materials based on metavirogenomic analyses (2, 3). However, these analyses often do not clearly indicate their host organisms. This study reported many novel viruses in the culturable phytopathogenic fungus *R. cerealis* that are all believed to replicate in this fungus. Although some fungi, such as *S. sclerotiorum* (25) and R. solani (18, 19) have been reported to host a large number of mycoviruses, there are few examples of more than 12 genera and 39 viruses in a single strain, as reported in *R. cerealis*. Additionally, viral metagenomics-based methods often fail to yield complete viral sequences (i.e., terminal ends), and this is a limitation of the study. Obtaining terminal ends of viruses still requires the use of RACE (random amplification of cDNA ends) methods, but it is not practical to apply this approach to a large number of virus samples. Continued advancements in high-throughput sequencing technology are necessary to overcome this problem. Although virological data such as infectivity, morphology, and viral effects on their hosts for these viruses are still lacking, this study provides a resource for the development and utilization of these viruses.

Based on RPKM values, we estimated the accumulation level of myoviruses in fungal host cells. The results indicated that the accumulation level of some mycoviruses, such as mitoviruses, in host cells was very high (Table 2). Moreover, the evolutionary relationships among the many viruses coexisting within a single strain are of great interest. Highly similar sequences were rare among different viruses in the same strain, indicating that horizontal transfer or recombination of genome segments between different viruses was rare. At present, it is clear that most mitoviruses exist in the mitochondria of their hosts (9, 29), but the ecological niches of other viruses in host cells are not clear. Whether virus locations in different niches within fungal cells can reduce the occurrence of horizontal gene transfer among viruses requires further study.

In conclusion, this study expands our understanding of the mycoviral diversity in *R. cerealis*, particularly that of ssRNA viruses, to some extent. We demonstrated that a large number of mycoviruses can coexist in the same *R. cerealis* strain. This study provides a valuable resource for the subsequent utilization of mycoviruses and raises many new questions. We currently know very little about how these mycoviruses coexist in the same fungal cell, how they escape cytoplasmic RNA silencing by the host, and how horizontal gene transfer, recombination, and coevolution occur and differ between viruses.

## MATERIALS AND METHODS

### Fungal strains, total-RNA and dsRNA extraction, and Illumina sequencing.

Four *R. cerealis* strains, R0928, R0942, R1084, and R10125, were collected from Henan, Anhui, and Shandong provinces in China (23). Total RNA was extracted from fungal mycelium using the RNAprep Pure Plant Plus kit (Tiangen, China). rRNA was depleted using the Ribo-Zero kit (Epicentre, Madison, WI, USA). The viral dsRNA of these strains was extracted and prepared as previously described (23). Library preparation and Illumina sequencing were performed by Genepioneer Bio-Tech Co., Ltd. (China), for rRNA-depleted samples and by Shanghai Hanyu Bio-Tech Co., Ltd. (China), for dsRNA samples. Each strain was subjected to rRNA depletion RNA-Seq and dsRNA-Seq.

### Genome sequence assembly and confirmation.

Before viral genome assembly, we filtered the reads from the host fungal genome. Reads from the clean RNA-Seq data were mapped to the genome of *R. cerealis* strain R0301 (24) with Bowtie2 (39) and SAMtools (40), and unmatched reads were extracted and assembled *de novo* using SOAPdenovo2 v2.04 (41) and Geneious Prime 2022.1.1 (42). The contigs of viral origin were selected based on BLAST searches against the Nr database of the National Center for Biotechnology Information (NCBI). Viral contigs obtained from rRNA depletion RNA-Seq and dsRNA-Seq were combined into one total viral sequence database. Each viral contig was used as a query for a BLAST search against the total viral sequence database to identify contigs overlapping the extended region. For each virus of each strain, all identified contigs were assembled again using the Geneious program. After several iterations, the viral genomes were finally determined.

The number of reads covering the viral genomes was obtained by mapping the reads from each sequenced library on reference sequences with Bowtie2 (39) and SAMtools (40). Mapping results were displayed using the Geneious program. Based on these results, the RPKM value and depth of coverage (calculated as bases of all mapped reads/length of virus) for each virus were calculated. For viruses with a depth of coverage less than 20×, we designed specific primers, amplified the genome sequences by RT-PCR, and sequenced them to confirm the viral genomes.

### Genome structure and phylogeny analysis.

The ORFs in the viral genomes were predicted using the Geneious program. The conserved domains and similar sequences of the deduced amino acid sequences were searched using BLASTp in the NCBI Conserved Domain Database, and domain hits with expected values less than 0.01 were considered (http://www.ncbi.nlm.nih.gov/Structure/cdd/wrpsb.cgi) (E value < 0.01). The genome structure of the viruses was displayed using the Geneious program.

The genome sequences of the viruses used in phylogenetic analysis were obtained from GenBank. Some of them were selected based on BLAST searches using the novel viruses in this study as a query. Other viruses representing different taxa were selected from the 2022 ICTV taxonomy release available online at https://ictv.global/taxonomy. Multiple alignments of the amino acid sequences of the viral RdRp regions were performed using the MAFFT program (https://myhits.isb-sib.ch/cgi-bin/mafft) (43) in the Geneious program. ModelFinder (44) was used to select the best-fit model by using the Bayesian information criterion. Maximum-likelihood phylogenies were generated via ultrafast bootstrap analysis in which 1,000 replicates were inferred using IQ-TREE (45), performed with the PhyloSuite v1.2.2 program (46). The display, annotation, and management of phylogenetic trees were performed using the online Interactive Tree Of Life tool (https://itol.embl.de/).

### Data availability.

All of the new genome sequences of the viruses have been deposited in GenBase (https://ngdc.cncb.ac.cn/genbase/) and GenBank (https://www.ncbi.nlm.nih.gov/genbank/); accession numbers can be found in Table S1 in the supplemental material. The raw sequence data reported in this paper have been deposited in the Genome Sequence Archive (GSA; CRA010887) in the National Genomics Data Center, Beijing Institute of Genomics, Chinese Academy of Sciences/China National Center for Bioinformation, which are publicly accessible at https://ngdc.cncb.ac.cn, and have been deposited in NCBI (BioProject no. PRJNA973243).
