# Supplementary material for: Extreme Diversity of Mycoviruses Present in Single Strains of Rhizoctonia cerealis, the Pathogen of Wheat Sharp Eyespot
Source: Microbiol Spectr. 2023 Jul 12;11(4):e00522-23. doi: 10.1128/spectrum.00522-23 (PMC10433806; doi:10.1128/spectrum.00522-23)
Supplement: Supplemental file 1 — Table S1. Download spectrum.00522-23-s0001.docx, DOCX file, 0.04 MB [file spectrum.00522-23-s0001.docx]

Table S1. The accession number of the viruses in this study in GenBase and GenBank.

| **Viruses** | **Sequence length (nt)** | **Accession number** | |
| --- | --- | --- | --- |
|  |  | **GenBase** | **GenBank** |
| RcMBLV-0928–1 | 10725 | C_AA001139 | OQ999696 |
| RcMBLV-0928–2 | 8648 | C_AA001140 | OQ999679 |
| RcMBLV-0928–3 | 8626 | C_AA001141 | OQ999689 |
| RcALV-0928–1 | 8427 | C_AA001142 | OQ999729 |
| RcALV-0942–1 | 8402 | C_AA001143 | OQ999744 |
| RcALV-1084–1 | 8440 | C_AA001145 | OQ999770 |
| RcALV-10125–1 | 8414 | C_AA001144 | OQ999695 |
| RcBeLV-0928–1 | 12336 | C_AA001146 | OQ999674 |
| RcBeLV-1084–1 | 12282 | C_AA001149 | OQ999721 |
| RcBeLV-10125–1 | 12475 | C_AA001147 | OQ999709 |
| RcBeLV-10125–2 | 10701 | C_AA001148 | OQ999735 |
| RcTyLV-0928–1 | 7888 | C_AA001241 | OQ999675 |
| RcTyLV-0942–1 | 7903 | C_AA001238 | OQ999761 |
| RcTyLV-1084–1 | 7911 | C_AA001239 | OQ999713 |
| RcTyLV-10125–1 | 7892 | C_AA001240 | OQ999756 |
| RcEV-0928–1 | 20866 | GWHAOPX01000001 | MW788753 |
| RcEV-0928–2 | 20602 | GWHAOPY01000001 | MW788754 |
| RcEV-0928–3 | 21771 | GWHAOPZ01000001 | MW788755 |
| RcEV-0928–4 | 20927 | GWHAOQA01000001 | MW788756 |
| RcEV-0928–5 | 17345 | GWHAOQB01000001 | MW788757 |
| RcEV-0942–2 | 17824 | GWHAOQD01000001 | MW788759 |
| RcEV-0942–3 | 19710 | GWHAOQE01000001 | MW788760 |
| RcEV-0942–4 | 20901 | GWHAOQF01000001 | MW788761 |
| RcEV-1084–1 | 20773 | GWHAOQH01000001 | MW788763 |
| RcEV-1084–2 | 19974 | GWHAOQI01000001 | MW788764 |
| RcEV-1084–3 | 20896 | GWHAOQJ01000001 | MW788765 |
| RcEV-1084–4 | 20869 | GWHAOQK01000001 | MW788766 |
| RcEV-1084–5 | 19717 | GWHAOQL01000001 | MW788767 |
| RcEV-1084–6 | 17495 | GWHAOQM01000001 | MW788768 |
| RcEV-1084–8 | 16383 | GWHAOQO01000001 | MW788770 |
| RcEV-1084–9 | 15480 | GWHAOQP01000001 | MW788771 |
| RcEV-10125–2 | 17274 | GWHAOQR01000001 | MW788773 |
| RcEV-0942–1 | 17148 | GWHAOQC01000001 | MW788758 |
| RcEV-1084–7 | 17106 | GWHAOQN01000001 | MW788769 |
| RcEV-10125–1 | 17181 | GWHAOQQ01000001 | MW788772 |
| RcDMV-0928–1 | 3946 | C_AA001184 | OQ999716 |
| RcDMV-0928–2 | 3872 | C_AA001185 | OQ999692 |
| RcDMV-0928–3 | 3737 | C_AA001186 | OQ999747 |
| RcDMV-0928–4 | 3436 | C_AA001187 | OQ999755 |
| RcDMV-0928–5 | 2809 | C_AA001188 | OQ999752 |
| RcDMV-0928–6 | 2674 | C_AA001189 | OQ999771 |
| RcDMV-0942–1 | 3871 | C_AA001170 | OQ999704 |
| RcDMV-0942–2 | 3723 | C_AA001171 | OQ999767 |
| RcDMV-0942–3 | 3273 | C_AA001172 | OQ999727 |
| RcDMV-0942–4 | 3180 | C_AA001173 | OQ999766 |
| RcDMV-0942–5 | 2824 | C_AA001174 | OQ999762 |
| RcMV1 | 3149 | —— | KM517201 |
| RcDMV-1084–2 | 3787 | C_AA001175 | OQ999743 |
| RcDMV-1084–3 | 3948 | C_AA001176 | OQ999717 |
| RcDMV-1084–4 | 3630 | C_AA001177 | OQ999738 |
| RcDMV-1084–5 | 3244 | C_AA001178 | OQ999765 |
| RcDMV-1084–6 | 2830 | C_AA001179 | OQ999678 |
| RcDMV-10125–1 | 4004 | C_AA001180 | OQ999690 |
| RcDMV-10125–2 | 3789 | C_AA001181 | OQ999706 |
| RcDMV-10125–3 | 3347 | C_AA001182 | OQ999782 |
| RcDMV-10125–4 | 2848 | C_AA001183 | OQ999711 |
| RcBYV-0928–1 | 13512 | C_AA001162 | OQ999780 |
| RcBYV-0928–2 | 13459 | C_AA001163 | OQ999728 |
| RcBYV-0928–3 | 13450 | C_AA001164 | OQ999749 |
| RcBYV-0928–4 | 13389 | C_AA001165 | OQ999775 |
| RcBYV-0928–5 | 12874 | C_AA001166 | OQ999745 |
| RcBYV-0928–6 | 12868 | C_AA001167 | OQ999724 |
| RcBYV-0928–7 | 12147 | C_AA001168 | OQ999764 |
| RcBYV-0928–8 | 11774 | C_AA001169 | OQ999778 |
| RcBYV-0942–1 | 13071 | C_AA001150 | OQ999774 |
| RcBYV-0942–2 | 13038 | C_AA001151 | OQ999769 |
| RcBYV-0942–3 | 12859 | C_AA001152 | OQ999707 |
| RcBYV-1084–1 | 13394 | C_AA001153 | OQ999737 |
| RcBYV-1084–2 | 13000 | C_AA001154 | OQ999734 |
| RcBYV-1084–3 | 13026 | C_AA001155 | OQ999772 |
| RcBYV-1084–4 | 12981 | C_AA001156 | OQ999758 |
| RcBYV-1084–5 | 12912 | C_AA001157 | OQ999731 |
| RcBYV-10125–1 | 13531 | C_AA001158 | OQ999781 |
| RcBYV-10125–2 | 13528 | C_AA001159 | OQ999700 |
| RcBYV-10125–3 | 12997 | C_AA001160 | OQ999736 |
| RcBYV-10125–4 | 12849 | C_AA001161 | OQ999746 |
| RcLeV-0928–1 | 8771 | C_AA001193 | OQ999719 |
| RcLeV-0928–2 | 7080 | C_AA001194 | OQ999776 |
| RcLeV-0928–3 | 7016 | C_AA001195 | OQ999681 |
| RcLeV-0928–4 | 7011 | C_AA001196 | OQ999726 |
| RcPhV-0928–1 | 11394 | C_AA001233 | OQ999702 |
| RcPhV-0928–2 | 9856 | C_AA001234 | OQ999730 |
| RcPhV-0928–3 | 6445 | C_AA001235 | OQ999733 |
| RcPhV-0942–1 | 9478 | C_AA001225 | OQ999691 |
| RcPhV-0942–2 | 6457 | C_AA001226 | OQ999687 |
| RcPhV-1084–1 | 10613 | C_AA001227 | OQ999750 |
| RcPhV-1084–2 | 9477 | C_AA001228 | OQ999753 |
| RcPhV-1084–3 | 9378 | C_AA001229 | OQ999705 |
| RcPhV-10125–1 | 11079 | C_AA001230 | OQ999768 |
| RcPhV-10125–2 | 10547 | C_AA001231 | OQ999763 |
| RcPhV-10125–3 | 4624 | C_AA001232 | OQ999714 |
| RcOCV-0928–1 | 2149 (RNA1) | C_AA001217 | OQ999697 |
|  | 1778 (RNA2) | C_AA001222 | OQ999760 |
| RcOCV-0928–2 | 2222 (RNA1) | C_AA001219 | OQ999759 |
|  | 1750 (RNA2) | C_AA001220 | OQ999699 |
| RcOCV-0928–3 | 2285 (RNA1) | C_AA001221 | OQ999710 |
|  | 1789 (RNA2) | C_AA001218 | OQ999680 |
| RcOCV-0928–4 | 2252 (RNA1) | C_AA001223 | OQ999739 |
|  | 1767 (RNA2) | C_AA001224 | OQ999740 |
| RcOCV-0942–1 | 2238 (RNA1) | C_AA001197 | OQ999757 |
|  | 1773 (RNA2) | C_AA001198 | OQ999718 |
| RcOCV-0942–2 | 2148 (RNA1) | C_AA001199 | OQ999773 |
|  | 1741 (RNA2) | C_AA001202 | OQ999742 |
| RcOCV-0942–3 | 2192 (RNA1) | C_AA001201 | OQ999693 |
|  | 1750 (RNA2) | C_AA001200 | OQ999683 |
| RcOCV-1084–1 | 2230 (RNA1) | C_AA001203 | OQ999694 |
|  | 1815 (RNA2) | C_AA001206 | OQ999712 |
| RcOCV-1084–2 | 2225 (RNA1) | C_AA001205 | OQ999673 |
|  | 1722 (RNA2) | C_AA001208 | OQ999741 |
| RcOCV-1084–3 | 2154 (RNA1) | C_AA001207 | OQ999701 |
|  | 1872 (RNA2) | C_AA001204 | OQ999777 |
| RcOCV-1084–4 | 2015 (RNA1) | C_AA001209 | OQ999725 |
|  | 1661 (RNA2) | C_AA001210 | OQ999684 |
| RcOCV-10125–1 | 2239 (RNA1) | C_AA001211 | OQ999703 |
|  | 1732 (RNA2) | C_AA001216 | OQ999748 |
| RcOCV-10125–2 | 2238 (RNA1) | C_AA001213 | OQ999688 |
|  | 1749 (RNA2) | C_AA001214 | OQ999751 |
| RcOCV-10125–3 | 2163 (RNA1) | C_AA001215 | OQ999682 |
|  | 1774 (RNA2) | C_AA001212 | OQ999723 |
| RcZHV-0928–1 | 13262 | C_AA001247 | OQ999708 |
| RcZHV-0928–2 | 13292 | C_AA001248 | OQ999686 |
| RcZHV-0942–1 | 13274 | C_AA001242 | OQ999732 |
| RcZHV-1084–1 | 13292 | C_AA001243 | OQ999715 |
| RcZHV-10125–1 | 14723 | C_AA001244 | OQ999720 |
| RcZHV-10125–2 | 12321 | C_AA001245 | OQ999685 |
| RcZHV-10125–3 | 13337 | C_AA001246 | OQ999698 |
| RcHV-0928–1 | 20697 | C_AA001192 | OQ999779 |
| RcHV-0942–1 | 20803 | C_AA001190 | OQ999677 |
| RcHV-1084–1 | 20713 | C_AA001191 | OQ999676 |
| RcPuV-1084–1 | 6728 | C_AA001236 | OQ999754 |
| RcPuV-10125–1 | 6641 | C_AA001237 | OQ999722 |
